# Are Pre and Postoperative Platelet to Lymphocyte Ratio and Neutrophil to Lymphocyte Ratio Associated with Early Postoperative AKI Following CABG?

**DOI:** 10.21470/1678-9741-2017-0164

**Published:** 2018

**Authors:** Hakan Parlar, Hüseyin Şaşkın

**Affiliations:** 1 Department of Cardiovascular Surgery, Derince Training and Research Hospital, Kocaeli, Turkey.

**Keywords:** Coronary Artery Bypass, Acute Kidney Injury, Biomarkers, Inflammation

## Abstract

**Objective:**

In this study, we investigated the role of two of the recent biomarkers of inflammation on the development of acute kidney injury in the early postoperative period of isolated coronary artery bypass grafting.

**Methods:**

Three hundred and eleven patients, who underwent isolated coronary artery bypass grafting with cardiopulmonary bypass by the same surgery team in our clinic between May 2010 and October 2014, who had a preoperative serum creatinine level lower than 1.5 mg/dl were included in the study. These patients' records were reviewed retrospectively. The diagnosis of acute kidney injury was performed according to the Kidney Disease Improving Global Outcomes 2012 Acute Kidney Injury Guideline criteria. Patients who developed acute kidney injury in the early postoperative period were classified as Group-1 (n=62) and the patients with normal postoperative renal functions were classified as Group-2 (n=249). The demographic data, body mass index, comorbidities, hematologic/biochemical profiles, preoperative ejection fraction, blood transfusion history, and operative data of the groups were compared. Univariate analyses were performed to determine significant clinical factors, and multiple logistic regression analyses were subsequently done to determine independent predictors of acute kidney injury.

**Results:**

Sixty-two (19.9%) patients developed acute kidney injury during the first 72 hours postoperatively. Multivariate logistic regression analyses revealed preoperative increased creatinine (*P*=0.0001), C-reactive protein (*P*=0.02), neutrophil-lymphocyte ratio (*P*=0.04) and platelet-lymphocyte ratio (*P*=0.002); increased postoperative first day leukocyte count (*P*=0.03), C-reactive protein levels (*P*=0.02), neutrophil-lymphocyte ratio (*P*=0.002), platelet-lymphocyte ratio (*P*=0.01) and increased intubation time (*P*=0.006) as independent predictors of early postoperative acute kidney injury in patients who underwent isolated coronary artery bypass grafting.

**Conclusion:**

The preoperative and postoperative increased levels of neutrophil-lymphocyte ratio and platelet-lymphocyte ratio which can be calculated by simple methods from routine blood analysis showed us that these parameters are independent biomarkers directly related to development of acute kidney injury in the early postoperative period.

**Table:** 

Abbreviations, acronyms & symbols				
ACT	= Activated clotting time		LIMA	= Left internal mammary artery
AKI	= Acute kidney injury		LVEF	= Left ventricular ejection fraction
AUC	= Area under curve		NLR	= Neutrophil to lymphocyte ratio
CABG	= Coronary artery bypass grafting		PCI	= Percutanous coronary intervention
CRP	= C-reactive protein		PLR	= Platelet to lymphocyte ratio
CPB	= Cardiopulmonary bypass		ROC	= Receiver operating curve
ECG	= Electrocardiography		RRT	= Renal replacement therapy
KDIGO	= Kidney Disease Improving Global Outcomes		STEMI	= ST-elevated myocardial infarction
LAD	= Left anterior descending artery		WBC	= White blood cell

## INTRODUCTION

Acute kidney injury (AKI) is one of the frequent pathological conditions following cardiac surgery which causes prolonged hospital and intensive care unit stay, higher health costs and mortality^[^^1^^]^. The incidence of AKI after cardiac surgery was reported as 5-30% and 1-2% of these patients required renal replacement therapy (RRT)^[^^2^^]^. AKI is a complicated pathology in a broad clinic spectrum from minimally increased serum creatinine levels to anuric status^[^^3^^]^. Multiple factors have been implicated as contributors to postoperative AKI including advanced age, female gender, chronic kidney disease, time delay between heart catheterization and surgery, aortic cross clamping time, duration of cardiopulmonary bypass (CPB) time, differences in the preoperative and intraoperative mean arterial pressures and blood transfusion following surgery^[^^3^^-^^5^^]^.

Neutrophil to lymphocyte ratio (NLR) and platelet to lymphocyte ratio (PLR) are recently described clinical parameters which can be found by detailed analysis of hemogram. There are many disorders with severe prognosis including peripheral vascular diseases, coronary artery disease, some of the malignancies of hepatobiliary and gynecological systems which are associated with elevated PLR levels. Elevated NLR levels have been demonstrated in systemic inflammation, cardiovascular disorders and gastrointestinal cancers^[^^6^^-^^8^^]^.

There have been no reports that focused on the association between the biomarkers NLR and PLR and AKI following coronary artery bypass grafting (CABG) in the recent literature. The aim of this study is to indicate the relationship between values of NLR and PLR and the development of AKI in patients in the early postoperative period of isolated CABG.

## METHODS

The medical records of a total of 506 patients who underwent isolated CABG between May 2010 and October 2014 were reviewed retrospectively. The operations were performed at the same center by the same surgery team; 311 patients who had serum creatinine levels of < 1.5 mg/dl who underwent isolated CABG with CPB were included in the study. The diagnosis of AKI was made by comparison of the baseline and postoperative serum creatinine levels to determine the presence of a predefined significant change based on the Kidney Disease Improving Global Outcomes (KDIGO) definition (increase in serum creatinine by ≥ 0.3 mg/dl within 48 hours of surgery or increase in serum creatinine to ≥ 1.5 times of baseline within 3 days of cardiac surgery)^[^^9^^]^.

The diagnosis of AKI was based on the highest serum creatinine concentration measured during the first 3 days after surgery compared with the baseline serum creatinine concentration, defined as the last concentration measured before surgery. Urine output was not used to define AKI, because it might be altered by diuretics administered during anaesthesia and CPB. Patients who developed AKI in the early postoperative period were classified as Group-1 (n=62) and the patients with normal postoperative renal functions were classified as Group-2 (n=249).

AKI was also staged for severity according to the following KDIGO criteria:

- Stage 1 - increase in serum creatinine by ≥ 0.3 mg/dl or 1.5 to 1.9 times of baseline,
- Stage 2 - increase in serum creatinine 2.0 to 2.9 times of baseline,
- Stage 3 - increase in serum creatinine 3.0 or more times of baseline or an increase in serum creatinine by ≥ 4.0 mg/dl or initiation of RRT^[^^10^^]^.

We excluded the patients previously diagnosed with end-stage renal disease who were on dialysis. The patients who had peripheral arterial disease, valvular heart disease, decompensated congestive heart failure, congenital cardiac disease, left ventricular systolic function disorder (left ventricular ejection fraction ≤ 30%), cerebrovascular disease in the last 30 days, renal impairment (serum creatinine > 1.5 mg/dl), chronic obstructive pulmonary disease, malignancy, endocrinologic disorders (hypothyroidism, hyperthyroidism), systemic inflammatory diseases, hematological proliferative diseases, low hemoglobin levels (≤ 10 g/dl); patients with acute infections, emergency operations, patients who were reoperated due to hemodynamic instability or bleeding, patients who required intra-aortic balloon pump, patients who had an acute myocardial infarction and percutaneous coronary intervention in the last 30 days prior to operation, patients with a diagnosis of active or chronic autoimmune diseases, patients who had steroid treatment or chemotherapy, patients who were operated on beating heart or redo CABG were also excluded from the study. Additionally, patients for whom data such as serum creatinine levels or urine output were missing, were excluded.

The demographic and clinical data of the patients were obtained by using the software system of the hospital for records and archives to investigate the patient files, epicrisis, operation notes and laboratory results. Age, gender, smoking history, diabetes, hypertension, hyperlipidemia, left ventricular ejection fraction (LVEF), preoperative and postoperative laboratory parameters (hemoglobin levels; leukocyte, platelet, lymphocyte and neutrophil counts; fasting blood glucose levels; serum creatinine and urea), operation information, the number of grafts used, duration of CPB and aortic cross clamping, amount of blood products used and length of stay in the intensive care unit and hospital were recorded.

Hypertension was accepted as a blood pressure of ≥ 140/90 mmHg or the use of antihypertensive drugs; smoking was accepted positive if the patient had not quitted smoking for the last one year. Diabetes was accepted as fasting blood glucose ≥ 126 mg/dl or the use of antidiabetic drugs, hyperlipidemia was accepted as total cholesterol > 220 mg/dl and LDL-cholesterol > 130 mg/dl or the use of antihyperlipidemic drugs.

Approximately 5 to 7 ml of venous blood samples were placed into a sterile tube with EDTA. Hematologic parameters were calculated by an automated blood count device (Abbott CELL-DYN 3700; Abbott Laboratory, Abbott Park, Illinois, USA) following a waiting time of one hour. PLR was calculated by dividing the number of platelets to the number of lymphocytes. NLR was calculated by dividing the number of neutrophils to the number of lymphocytes.

All patients were transferred to intensive care unit intubated postoperatively. They were extubated following onset of spontaneous breathing and normalization of orientation and cooperation if the hemodynamic and respiratory functions were appropriate. If there was no contraindication, 50 mg/day of metoprolol was started orally to all patients following the first postoperative day. The diagnosis of postoperative atrial fibrillation was made by standard 12 derivation electrocardiography (ECG).

Written informed consent form was obtained from all the patients included in the study. This study complied with the Declaration of Helsinki and was carried out following approval of Ethics Committee for Clinical Trials of Medical Faculty of Kocaeli University.

### Operative Technique

All patients were operated with median sternotomy under general anaesthesia and CPB with aortic and venous cannulations following systemic heparin administration (300 IU/kg). Activated clotting time (ACT) was maintained over 450 seconds during the operations. Standard CPB circuit and surgical management were used. Antegrade hypothermic and hyperkalemic blood cardioplegia was applied to all patients. Surgery was performed under moderate systemic hypothermia (28-30ºC). Cardiopulmonary bypass flow was maintained 2.2-2.5 l/min/m^2^, mean perfusion pressure was maintained between 50 and 80 mmHg, hematocrit level was maintained between 20 to 25 percent during CPB. For the coronary bypass operations, the left internal mammary artery (LIMA) was preferred for the arterial graft for left anterior descending artery (LAD) revascularization, whereas saphenous venous grafts were used for the other vessels. Distal anastomoses were done during aortic cross clamping period and proximal anastomoses were done on beating heart onto the ascending aorta using lateral clamp.

### Statistical Analysis

Statistical analysis was performed using the SPSS software version 13.0 (SPSS Inc, Chicago, IL, USA). Among the data measured, those showing normal distribution were expressed as mean ± standard deviation, those not showing normal distribution were expressed as median (minimum-maximum). The data obtained by counting were given as percentages (%). Among the data measured, the normality of distribution was evaluated by histogram or Kolmogorov-Smirnov test, the homogenity of distribution was evaluated by Levene's test for equality of variance. Among the data measured, the difference between the groups was evaluated by Student's t-test in normal and homogenous distribution and by Mann-Whitney U test in distribution which is not normal and homogeneous. Among the data obtained by counting, the differences between the groups were evaluated by parametric or non-parametric Pearson Chi-Square test or Fisher's Exact test according to the distribution being parametric or not. The effects of the risk factors suggested to be influential on the early period AKI were studies through the univariate logistic regression analysis. The multiple effects of the risk factors which are influential or which are suggested to be influential in predicting the early period AKI as a result of the univariate statistical analysis were studied through the retrospective selective multivariate logistic regression analysis. The odds ratio, the 95% confidence interval and the significant level for each of the risk factors, was found. The sensitivity and the specificity of PLR and NLR in predicting the early period AKI via receiver operating curve (ROC) were computed and results were found as statistically significant for *P*<0.05.

## RESULTS

The demographic characteristics and clinical data of the patients were summarized in Table 1. There were no differences between the two groups in terms of demographic or clinical data.

**Table 1:** Comparison of patient's characteristics between the 2 groups.

Patient's Characteristics	Group I AKI (n=62)	Group II Non-AKI (n=249)	*P* value
Age (years) (median; min-max)	62 (35-83)	60 (33-82)	0.14**
Male (%)	46 (74.2%)	189 (75.9%)	0.78*
Female (%)	16 (25.8%)	60 (24.1%)	
BMI (kg/m^2^) (median; min-max)	28.5 (19.6-37.4)	27.3 (19.0-39.4)	0.11**
Hypertension (%)	41 (66.1%)	152 (61.0%)	0.46*
Diabetes mellitus (%)	29 (46.8%)	85 (34.1%)	0.07*
Smoking (%)	29 (46.8%)	93 (37.3%)	0.17*
Hyperlipidaemia (%)	26 (41.9%)	116 (46.6%)	0.51*
Ejection fraction (%) (median; min-max)	56.5 (30-68)	58 (30-67)	0.35**
History of atrial fibrillation (%)	7 (11.3%)	25 (10.0%)	0.77*
History of CVA (%)	2 (3.2%)	6 (2.4%)	0.66*
History of MI (%)	14 (22.6%)	51 (20.5%)	0.72*

* Pearson Chi-Square test or Fisher's Exact test

** Mann-Whitney U test

BMI=body mass index; CVA=cerebrovascular accident; MI=myocardial infarction

According to the KDIGO classification, 51.6% (n=32) of the patients were Stage-I, 38.7% (n=24) were Stage-II and 9.7% (n=6) were Stage-III. The preoperative blood analysis and hematological parameters of the patients were summarized in Table 2. Preoperative creatinine levels (*P*=0.0001), urea levels (*P*=0.0001), lymphocyte counts (*P*=0.04), neutrophil counts (*P*=0.03), PLR (*P*=0.0001), NLR (*P*=0.0001) and C-reactive protein (CRP) levels(*P*=0.0001) were significantly different between the groups.

**Table 2:** Preoperative blood results and hematological parameters of patients.

Preoperative blood results and hematological parameters	Group I AKI (n=62)	Group II Non-AKI (n=249)	*P* value
	Median (minimum-maximum)	Median (minimum-maximum)	
Preoperative hemoglobin (mg/dl)	13.9 (10.6-16.0)	13.5 (10.4-16.5)	0.24*
Preoperative hematocrit (%)	41.8 (30.6-48.9)	40.5 (30.5-48.9)	0.42*
Preoperative creatinine (mg/dl)	1.20 (0.60-1.46)	0.81 (0.60-1.47)	0.0001*
Preoperative urea (mg/dl)	23 (12-47)	18 (10-39)	0.0001*
Preoperative leukocyte count (x10^3^/µL)	8.4 (5.2-11.4)	8.0 (3.4-11.8)	0.52*
Preoperative platelet count (x10^3^/µL)	290 (156-444)	278 (145-445)	0.13*
Preoperative CRP (mg/L)	1.12 (0.36-4.58)	0.49 (0.16-4.71)	0.0001*
Preoperative lymphocyte count (x10^3^/µL)	1.8 (1.0-4.9)	2.2 (0.9-4.9)	0.04*
Preoperative neutrophil count (x10^3^/µL)	5.9 (2.8-9.6)	5.0 (1.4-9.7)	0.003*
Preoperative NLR	3.3 (1.2-8.9)	2.3 (0.6-8.0)	0.0001*
Preoperative PLR	154.5 (84.7-191.7)	122.9 (80.0-199.3)	0.0001*

* Mann-Whitney U test

CRP=C-reactive protein; NLR=neutrophil to lymphocyte ratio; PLR=platelet to lymphocyte ratio

The early postoperative blood analysis and hematological parameters of the patients were summarized in Table 3. Leukocyte counts (*P*=0.0001) in the first and third postoperative days; CRP levels (*P*=0.0001), lymphocyte counts (*P*=0.0001), neutrophil counts (respectively *P*=0.009, *P*=0.03, *P*=0.0001), NLR (*P*=0.0001) and PLR (*P*=0.0001) in the first, third and seventh postoperative days were significantly different between the groups.

**Table 3:** Early postoperative blood results and hematological parameters of patients.

Early postoperative period blood results and hematological parameters		Group I AKI (n=62)	Group II Non-AKI (n=249)	*P* value
		Median (minimum-maximum)	Median (minimum-maximum)	
Postoperative hemoglobin (mg/dl)	1 day	8.8 (7.5-11.4)	9.0 (7.4-12.6)	0.50*
Postoperative hematocrit (%)	1 day	28.0 (24.7-36.2)	28.5 (24.3-39.0)	0.52*
Postoperative leukocyte count (x10^3^/µL)	1 day	15.1 (7.6-21.1)	12.5 (4.7-32.3)	0.0001*
	3 days	13.3 (6.8-26.4)	11.1 (4.6-21.1)	0.0001*
	7 days	10.3 (7.0-23.3)	9.8 (4.3-22.1)	0.30*
Postoperative platelet count (x10^3^/µL)	1 day	162 (77-331)	177 (71-350)	0.17*
	3 days	165 (65-320)	167 (70-327)	0.50*
	7 days	214 (99-463)	231 (100-408)	0.17*
Postoperative CRP (mg/L)	1 day	32.1 (15.2-44.1)	26.7 (15.2-39.4)	0.0001*
	3 days	43.9 (36.5-63.3)	41.0 (30.5-48.9)	0.0001*
	7 days	9.5 (7.4-19.9)	8.9 (4.8-12.6)	0.0001*
Postoperative lymphocyte count (x10^3^/µL)	1 day	1.1 (0.4-2.2)	1.5 (0.6-2.6)	0.0001*
	3 days	1.1 (0.4-2.7)	1.3 (0.6-3.3)	0.0001*
	7 days	1.4 (0.6-3.2)	1.9 (0.9-3.7)	0.0001*
Postoperative neutrophil count (x10^3^/µL)	1 day	8.1 (3.9-11.7)	7.3 (2.8-9.8)	0.009*
	3 days	7.7 (4.0-10.8)	7.4 (3.0-10.8)	0.03*
	7 days	8.1 (3.5-11.0)	6.5 (1.4-10.5)	0.0001*
Postoperative NLR	1 day	7.9 (4.5-10.2)	5.1 (2.1-9.9)	0.0001*
	3 days	7.8 (3.5-10.7)	5.4 (1.4-10.6)	0.0001*
	7 days	5.6 (1.6-10.6)	3.3 (0.5-8.6)	0.0001*
Postoperative PLR	1 day	156.6 (108.2-198.9)	124.7 (72.2-188.6)	0.0001*
	3 days	152.6 (81.5-196.3)	120.9 (69.4-172.5)	0.0001*
	7 days	155.7 (82.5-197.9)	120.0 (68.7-169.2)	0.0001*

* Mann-Whitney U test

CRP=C-reactive protein; NLR=Neutrophil to lymphocyte ratio; PLR=platelet to lymphocyte ratio

The intraoperative and postoperative data of the patients were shown in Table 4. Intubation time (*P*=0.0001), length of stay in the intensive care unit (*P*=0.0001), use of inotropic support (*P*=0.02) and total length of stay in the hospital (*P*=0.0001) were significantly different between the groups. We performed LIMA anastomosis to LAD in 57 (91.9%) patients in Group-1 and in 241 (96.8%) patients in Group-2.

**Table 4:** Intraoperative and postoperative data of the patients.

Characteristics	Group I AKI (n=62)	Group II Non-AKI (n=249)	*P* value
	Median (minimum-maximum)	Median (minimum-maximum)	
Aortic cross clamp time (minutes)	51 (23-84)	51 (21-76)	0.73**
Cardiopulmonary bypass time (minutes)	84.5 (47-114)	84 (42-110)	0.50**
Number of distal anastomoses	3 (1-5)	3 (1-5)	0.68**
Amount of drainage (ml)	300 (200-1350)	300 (150-1250)	0.08*
Intubation time (hours)	8 (3-16)	5 (3-21)	0.0001**
Stay in the intensive care unit (hours)	37.5 (21-87)	21 (17-66)	0.0001**
Total duration of hospital stay (days)	8 (5-14)	5 (5-10)	0.0001**
Use of inotropic support (%)	16 (25.8%)	33 (13.3%)	0.02*
Use of blood products (%)	31 (50.0%)	117 (47.0%)	0.67*

* Pearson Chi-Square test or Fisher's Exact test

** Mann-Whitney U test

The results of univariate and multivariate regression analyses of patients who developed AKI in the early postoperative period were shown in Table 5. In the multivariate analysis of the variables which were found statistically significant in univariate analysis associated with postoperative AKI, increased serum creatinine (*P*=0.0001), CRP levels (*P*=0.02), PLR (*P*=0.002) and NLR (*P*=0.04) in the preoperative period, increased leukocyte counts (*P*=0.03), CRP levels (*P*=0.02), NLR (*P*=0.002) and PLR (*P*=0.01) in the first postoperative day and prolonged intubation time (*P*=0.006) were found as independent predictors of early postoperative AKI.

**Table 5:** Univariate and multivariate regression analyses of preoperative, operative and early postoperative risk factors for postoperative AKI.

Variables	Postoperative AKI			
	Unadjusted OR (95% CI)	*P*	Adjusted OR (95% CI)	*P*
Gender	0.91 (0.48-1.73)	0.87	__	__
Age	1.02 (0.99-1.05)	0.16	__	__
Ejection fraction (%)	0.98 (0.95-1.01)	0.15	__	__
Diabetes mellitus	1.70 (0.97-2.98)	0.17	__	__
Hypertension	1.25 (0.70-2.24)	0.46	__	__
Hyperlipidaemia	0.83 (0.47-1.45)	0.51	__	__
Smoking	1.47 (0.84-2.58)	0.18	__	__
BMI	1.05 (0.98-1.12)	0.20	__	__
Preoperative creatinine (mg/dl)	2464.06 (383.62-15827.07)	0.0001	4097.94 (52.93-317299.77)	0.0001
Preoperative urea (mg/dl)	1.12 (1.07-1.17)	0.0001	1.05 (0.94-1.18)	0.37
Preoperative platelet (x10^3^/µL)	1.00 (0.99-1.01)	0.12	__	__
Preoperative CRP (mg/l)	3.17 (2.12-4.75)	0.0001	2.71 (1.16-6.35)	0.02
Preoperative hemoglobin (mg/dl)	1.13 (0.92-1.37)	0.24	__	__
Preoperative leukocyte (x10^3^/µL)	0.99 (0.98-1.00)	0.44	__	__
Preoperative NLR	1.76 (1.38-2.23)	0.0001	0.45 (0.22-0.95)	0.04
Preoperative PLR	1.05 (1.03-1.07)	0.0001	1.06 (1.02-1.10)	0.002
Postoperative first day platelet (x10^3^/µL)	1.32 (1.08-1.62)	0.007	1.00 (0.99-1.01)	0.20
Postoperative first day hemoglobin (mg/l)	0.90 (0.70-1.16)	0.42	__	__
Postoperative first day leukocyte (x10^3^/µL)	0.97 (0.92-1.02)	0.0001	1.01(0.99-1.03)	0.03
Postoperative first day CRP (mg/l)	1.25 (1.17-1.33)	0.0001	1.19 (1.02-1.36)	0.02
Postoperative first day NLR	2.46 (1.97-3.07)	0.0001	3.04 (1.51-6.10)	0.002
Postoperative first day PLR	1.09 (1.07-1.12)	0.0001	1.06 (1.01-1.10)	0.01
Aortic cross clamp time	1.01 (0.99-1.03)	0.48	__	__
Number of distal anastomoses	1.11 (0.82-1.49)	0.50	__	__
CPB time	1.01 (0.99-1.03)	0.37	__	__
Intubation time	1.44 (1.27-1.63)	0.0001	1.64 (1.15-2.33)	0.006
Use of blood products	1.13 (0.65-1.97)	0.67	__	__
Use of inotropic support	2.28 (1.16-4.48)	0.02	0.31(0.08-2.29)	0.17
Amount of drainage	1.00 (0.99-1.01)	0.18	__	__

AF=atrial fibrillation; BMI = body mass index; CPB=cardiopulmonary bypass; CRP=C-reactive protein; NLR=neutrophil to lymphocyte ratio; PLR=platelet to lymphocyte ratio

**Fig. 1:**
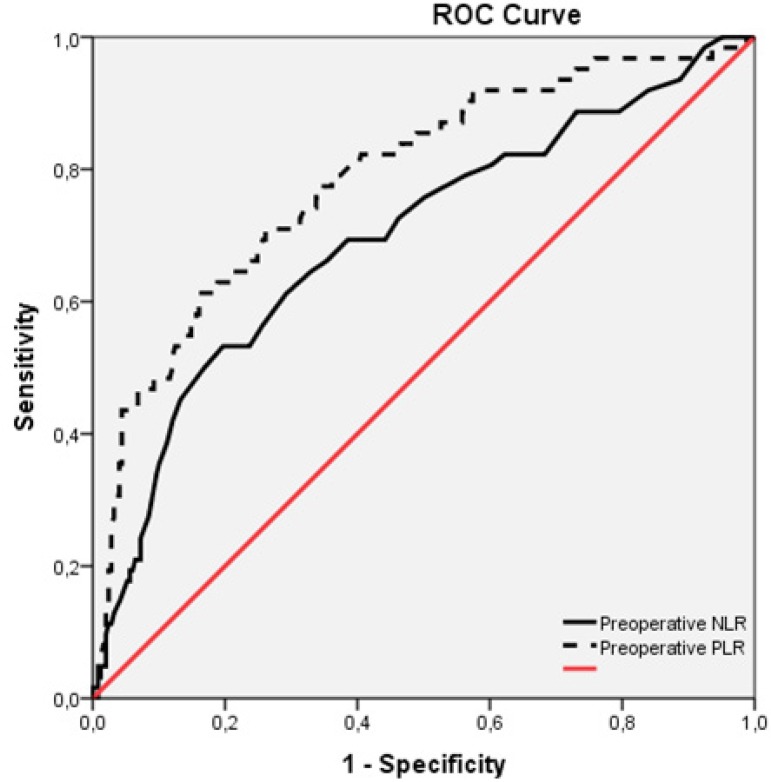
ROC curve analysis or preoperative PLR and NLR regarding occurrence of postoperative AKI.

The ROC curves for preoperative NLR and PLR were in connection with postoperative AKI following isolated CABG (Figure 1). The area under curve (AUC) for the preoperative NLR was 0.697 (95% CI 0.620- 0.774; *P*=0.0001). Using a cut-off value of 2.65, the preoperative PLR predicted postoperative AKI with a sensitivity of 66.1% and specificity of 64.7%. The AUC for the preoperative PLR was 0.786 (95% CI 0.719- 0.851; =0.0001). Using a cut-off value of 136.85, the preoperative PLR predicted postoperative AKI with a sensitivity of 71.0% and specificity of 70.7%.

**Fig. 2:**
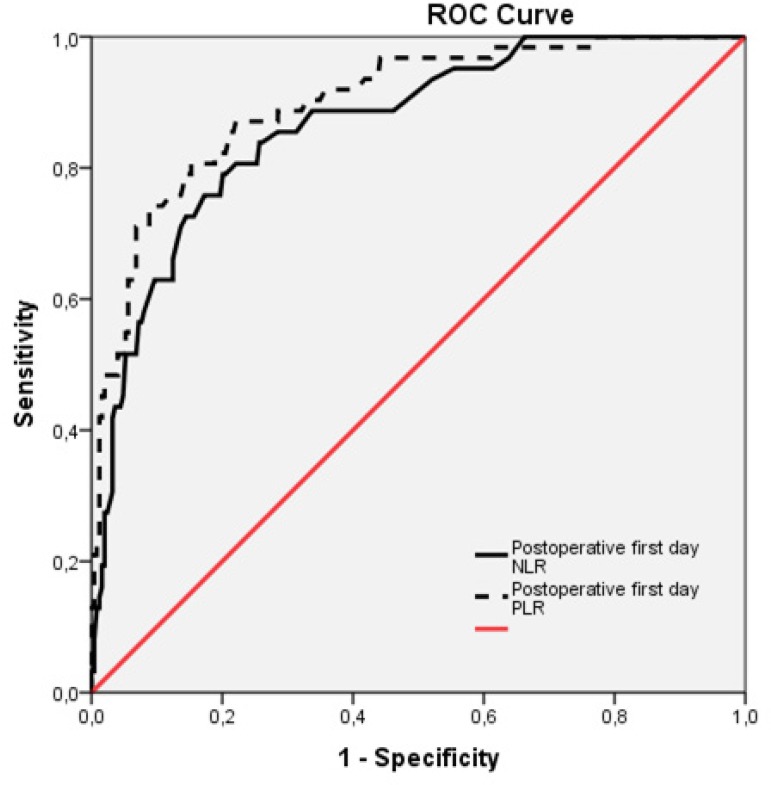
ROC curve analysis or postoperative first day PLR and NLR regarding occurrence of postoperative AKI.

The ROC curves for the postoperative first day NLR and PLR were in connection with postoperative AKI following isolated CABG (Figure 2). The AUC for the preoperative NLR was 0.864 (95% CI 0.815- 0.914; *P*=0.0001). Using a cut-off value of 6.15, the preoperative PLR predicted postoperative AKI with a sensitivity of 80.6% and specificity of 77.9%. The AUC for the preoperative PLR was 0.899 (95% CI 0.856- 0.943; *P*=0.0001). Using a cut-off value of 139.25, the preoperative PLR predicted postoperative AKI with a sensitivity of 82.3% and specificity of 81.1%.

## DISCUSSION

In this study, increased preoperative and early postoperative NLR and PLR were associated with postoperative AKI following isolated CABG operation. To the best of our knowledge; this is the first study to evaluate the association of NLR and PLR with early postoperative AKI after CABG operation.

AKI is a common postoperative complication of cardiac surgery, associated with a prolonged hospital stay and increased morbidity and mortality, even for patients who do not progress to renal failure. The incidence of AKI following cardiac surgery is 5-30%^[^^2^^]^. In our study, we have identified the occurrence of AKI after CABG as 19.9%.

The pathophysiology of AKI is complex and multifactorial, and the locus of injury is typically the tubular cells and involves toxins (exogenous and endogenous), inflammation, ischemia-reperfusion injury, neurohormonal activation, metabolic factors and oxidative stress^[^^11^^]^. Another cause of AKI following cardiac surgery could be a pro-inflammatory event such as operative trauma, blood exposed to the artificial surface of the CPB circuit or ischaemia-reperfusion injury^[^^2^^]^. C-reactive protein is a marker of inflammation that is extensively used in clinical practice. Recently, several prospective clinical studies have shown that modest elevations in baseline CRP levels predict future cardiovascular events^[^^12^^]^. Shacham et al.^[^^13^^]^ reported >9 mg/l of high sensitive CRP levels as an independent risk factor for AKI following primary percutanous interventions of the patients with ST-elevated myocardial infarction (STEMI). In our study, we found that the preoperative and early postoperative increased CRP levels are independent risk factors for the development of AKI in the early postoperative period.

Several inflammatory biomarkers including white blood cell (WBC) count, leukocyte subtypes, platelet, CRP, NLR and PLR have been demonstrated to be important prognostic predictors in various cardiovascular diseases^[^^14^^]^. Each WBC subtype, including neutrophils, lymphocytes, monocytes, and eosinophils, has a discrete role in inflammation, host defense, and repair^[^^15^^]^. Each of these components are one of the immunologic factors that play a pivotal role in most processes of organ damage and this role can apply to the issue of kidney damage^[^^16^^]^. In our study, we have determined that the blood leukocyte levels were significantly higher in the AKI group in the 1^st^ and 3^rd^ days postoperatively.

NLR is a new inflammatory marker which is associated with inflammation^[^^17^^]^. It has also been shown that NLR was associated with adverse events following CABG in stable and unstable angina pectoris. Ünal et al.^[^^18^^]^ have observed that preoperative NLR was correlated with mortality after CABG. Yilmaz et al.^[^^19^^]^ reported that NLR is superior to CRP and WBC, to predict the development of AKI in the patients with severe sepsis. In our study, we identified that the preoperative and postoperative NLR values were significantly high in patients with AKI.

To our knowledge, there have been no studies in the literature with patients who underwent isolated CABG with CPB, that investigated the relationship between the development of postoperative AKI and NLR as a biomarker, so we suppose that our findings are valuable. PLR is a recently defined hematological parameter which is associated with both aggregation and inflammation pathways, and it can be more valuable than either platelet or lymphocyte counts alone in predicting coronary artery disease^[^^20^^]^. PLR has been used to predict the prognosis of patients with different inflammatory and ischemic events in the literature^[^^21^^]^. Temiz et al.^[^^22^^]^ have observed elevated PLR to be associated with hospital mortality in patients with STEMI. Akin et al.^[^^23^^]^ reported in their study including 630 patients who underwent percutanous coronary intervention (PCI) after STEMI that the patients who had higher PLR values before the procedure developed contrast-induced AKI. In our study PLR values of AKI Group were significantly high.

### Limitations of the Study

A few limitations of our study deserve mention. This is a single centre retrospective study with a relatively small sample size. Although we have found some parameters associated with AKI, we could not draw a causal relationship.

## CONCLUSION

In our study, we observed that elevated NLR and PLR were independent predictors of early postoperative AKI following isolated CABG. NLR and PLR, which can easily be obtained from a simple whole blood count, may predict adverse events after CABG. Although we could not establish a causal relationship in this study, the results of this study may have some clinical implications if approved by large scale prospective studies.

**Table:** 

Authors' roles & responsibilities	
HP	Substantial contributions to the conception or design of the work; or the acquisition, analysis, or interpretation of data for the work; final approval of the version to be published
HŞ	Substantial contributions to the conception or design of the work; or the acquisition, analysis, or interpretation of data for the work; final approval of the version to be published
